# Book Reviews

**Published:** 1885-11

**Authors:** 


Œsterreichisch—Ungarische Viertlejahrsschrift fur Zahnheilkunde. Redacteur, Dr. Heinrich Schmid docent a. d. k. k., deutschen Universitat in Prag, Abt. M., 5, pr. Jahr., 8o., Wien, Jan., 1885. Quarterly.

Want of space has prevented our extending an earlier welcome to this excellent addition to the number of German dental periodicals, of which the second and third numbers only seem to have reached us. Original papers by Professor Dr. Ritter, Drs. Arkovy and Matrai, cases in practice, Transactions of the Austrian Dental Society, and of the Vienna Dental Society, together with extracts of cognate matter from the German Medical Journals make up an attractive list of contents, which should secure for the Austrian Journal a large circulation amongst our many reading fellow practitioners. We hope to return on some future occasion to the consideration of an article "On the Education in Dentistry at the Vienna University compared with that of Berlin and Leipzig.

---

Dental Surgery for Practitioners and Students, by Ashley W. Barrett M. B., London, M. R. C., L. D. S., Dental Surgeon to the London Hospital; pp. xii., 83, sm. 8o. P. Blakiston Son & Co., Philadelphia, 1885.

This is a neat volume, containing much useful information, but we think that it scarcely supplies exactly what is required by the medical student or practitioner who has not made or does not intend to make dentistry a specality. In the former case when consulted by a patient in great suffering, the duties of a surgeon should be limited to the application of an anodyne, or in cases of manifest necessity, the extraction of a tooth; in the latter case we conceive that the subject of Dental Surgery should receive a far more comprehensive treatment. The illustrations are numerous, appropriate and excellent. We are very glad to see an elementary work written by a gentleman of Mr. Barrett's eminence, and trust that the work will have a large circulation among dentists.

